# RNase E-dependent processing stabilizes MicX, a *Vibrio cholerae* sRNA

**DOI:** 10.1111/j.1365-2958.2007.05796.x

**Published:** 2007-07-01

**Authors:** Brigid M Davis, Matthew K Waldor

**Affiliations:** Channing Laboratory, Brigham and Women's Hospital, and Howard Hughes Medical Institute Boston, MA 02111, USA.

## Abstract

In *Vibrio cholerae*, bioinformatic approaches have been used to predict the locations of numerous small RNA (sRNA)-encoding genes, but biological roles have been determined for very few. Here, we describe the expression, processing and biological role of an sRNA (previously known as A10) that was identified through such analyses. We have renamed this sRNA MicX as, like the *Escherichia coli* sRNAs MicA, MicC and MicF, it regulates expression of an outer membrane protein (OMP). MicX appears to be a direct negative regulator of *vc0972*, which encodes an uncharacterized OMP, and *vc0620*, which encodes the periplasmic component of a peptide ABC transporter. Hfq is apparently not required for MicX's interactions with and regulation of these targets. The sequence encoding MicX overlaps with *vca0943*; however, primary transcripts of MicX are processed in an RNase E- and Hfq-dependent fashion to a shorter, still active and much more stable form consisting largely of the *vca0943* 3′ untranslated region. Our data suggest that processing of MicX enhances its effectiveness, and that sRNA cleavage is not simply a means to sRNA inactivation and clearance.

## Introduction

In recent years, it has become evident that the genomes of many bacteria encode a plethora of regulatory small RNAs (sRNAs) that enable post-transcriptional modulation of gene expression (reviewed in Gottesman, 2004). A few of these interact primarily with proteins; however, the majority act by base pairing with mRNAs. Binding between regulatory sRNAs and mRNA targets typically results in altered mRNA structure, stability and/or translation. For most sRNAs characterized to date, this interaction decreases the abundance of target mRNAs and consequently inhibits gene expression; however, sRNAs that can promote gene expression have also been identified. sRNA-dependent regulation contributes to cellular adaptation in response to numerous stimuli, including cold, high osmolarity, DNA damage, phosphosugar stress, iron limitation and oxidative stress (Altuvia *et al*., 1997; Repoila and Gottesman, 2001; Masse and Gottesman, 2002; Vanderpool and Gottesman, 2004; Vogel *et al*., 2004; Guillier and Gottesman, 2006).

The vast majority of sRNAs with defined targets are not genetically linked to their target genes; consequently, unlike most plasmid-encoded regulatory RNAs (Gerhart *et al*., 1998), there is no perfect or extensive sequence complementarity between sRNAs and their corresponding mRNAs. Instead, sRNA:mRNA binding is generally mediated by relatively short and imperfect regions of sequence complementarity (Gottesman, 2005). Perhaps as a result, many sRNAs have been found to be inactive in cells lacking the RNA-binding protein Hfq. Hfq binds to many sRNAs and their mRNA targets, and it can alter RNAs' secondary structures and promote RNA–RNA interactions (Moller *et al*., 2002a; Zhang *et al*., 2002; Moll *et al*., 2003; Geissmann and Touati, 2004; Rasmussen *et al*., 2005). Hfq also stabilizes numerous sRNAs, potentially by protecting them from degradation by RNase E, as Hfq and RNase E are thought to recognize similar sequences and structures (Moll *et al*., 2003). In several cases, it appears that sRNA overexpression can restore regulation of mRNA targets in the absence of Hfq (Sledjeski *et al*., 2001; Castillo-Keller *et al*., 2006).

Many sRNAs have been reported to bind near the 5′ ends of their mRNA targets, in particular to sequences overlapping with or adjacent to the Shine–Dalgarno sequence. Such sRNA:mRNA complex formation can prevent binding of ribosomes, thereby inhibiting translation and allowing rapid dowregulation of gene expression (Moller *et al*., 2002b; Geissmann and Touati, 2004; Rasmussen *et al*., 2005; Udekwu *et al*., 2005). In the absence of ribosome binding, mRNAs are also thought to be more vulnerable to cleavage by RNases, resulting in decreased transcript abundance (Yarchuk *et al*., 1991). Genetic and biochemical evidence suggests that both RNase E and RNase III can mediate cleavage of target mRNAs, and that degradation of mRNAs may be coupled to sRNA degradation (Masse *et al*., 2003; Vogel *et al*., 2004; Afonyushkin *et al*., 2005). However, it is not entirely clear why sRNA:mRNA pairing should promote sRNA degradation, particularly cleavage dependent upon RNase E, which is thought to principally recognize single-stranded regions. It should also be noted that not all sRNAs that bind to 5′ untranslated sequences inhibit gene expression or promote target degradation. For example, binding of both DsrA and RprA to regions upstream of the ribosome binding site (RBS) in *rpoS* mRNA promotes translation, apparently by preventing formation of mRNA secondary structures that occlude access to the RBS (Majdalani *et al*., 1998; 2002).

Small RNAs have been identified using a variety of approaches, including physical purification, metabolic labelling, and both fortuitous and systematic cloning and expression (reviewed in Gottesman, 2004). In the past few years, genome-wide bioinformatic analyses have also identified numerous candidate sRNA-encoding genes, many of which await experimental confirmation and/or characterization (Argaman *et al*., 2001; Rivas *et al*., 2001; Wassarman *et al*., 2001; Livny *et al*., 2005; 2006; Saetrom *et al*., 2005). In general, regulatory sRNAs appear to be conserved among closely related species but are not maintained in recognizable form in distantly related species, suggesting either recent emergence or rapid sequence divergence (Hershberg *et al*., 2003). Most characterized sRNAs are encoded by sequences that are not predicted to code for proteins, are transcribed from sRNA-specific promoters rather than as part of an operon, and are associated with rho-independent terminators. Several appear to be post-transcriptionally processed (Faubladier *et al*., 1990; Moll *et al*., 2003), but the biological significance of sRNA processing is largely unexplored.

In *Vibrio cholerae*, the cause of the diarrhoeal disease cholera, bioinformatic approaches have been used to predict the locations of numerous putative sRNA-encoding genes (Masse and Gottesman, 2002; Lenz *et al*., 2004; Livny *et al*., 2005). sRNA transcription has been confirmed for a subset of these, but biological roles have been identified for only a few (Lenz *et al*., 2004; Davis *et al*., 2005). A set of four redundant sRNAs have been shown to be essential for production of several *V. cholerae* virulence factors (Lenz *et al*., 2004), and characterization of a *V. cholerae hfq* mutant suggested that additional sRNAs are also required for *V. cholerae* growth within the intestine (Ding *et al*., 2004). In this work, we describe the expression, processing and biological role of the sRNA previously known as A10, which we identified using sRNAPredict, an algorithm that detects potential sRNA-encoding genes within intergenic sequences (Livny *et al*., 2005). Based on our observation that it regulates expression of an outer membrane protein (OMP), as do the *Escherichia coli* sRNAs MicA, MicC and MicF (Mizuno *et al*., 1984; Chen *et al*., 2004; Rasmussen *et al*., 2005; Udekwu *et al*., 2005), we have renamed it MicX. The primary transcript for MicX overlaps the 3′ end of *vca0943*; however, expression of MicX is controlled by a gene-specific promoter. MicX is processed by RNase E to a shorter form consisting largely of the *vca0943* 3′ untranslated region (UTR). Both forms have biological activity, but the processed form is significantly more stable (> 10×) and abundant. Processed forms of MicX are not detected in the absence of Hfq, although some regulation of mRNA targets by MicX still occurs in the absence of this protein. MicX appears to be a direct negative regulator of *vc0972*, which encodes an uncharacterized OMP, and *vc0620*, which encodes the periplasmic component of a peptide ABC transporter.

## Results

### The sRNA MicX overlaps with *vca0943* mRNA and is independently expressed

**Fig. 1:**
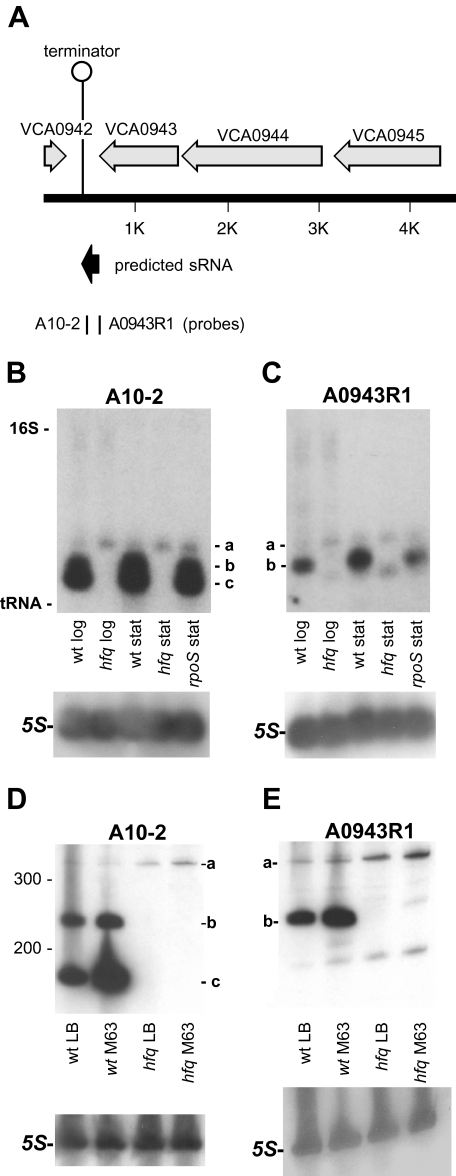
Expression of *micX*, an sRNA-encoding gene that overlaps with *vca0943* A. Schematic depiction of the *micX*-encoding region of the *V. cholerae* genome. The grey arrows represent predicted coding sequences, the black arrow shows the sRNA (MicX, formerly A10) predicted by bioinformatic analysis, and short lines show the positions of complimentary oligonucleotide probes used for Northern hybridizations. The probe A0943R1 is complementary to the final 10 codons of *vca0943.* The lollipop shows the position of a predicted rho-independent terminator for *vca0943*. B. Northern blot probed with oligonucleotide A10-2 (upper panel) or a control probe for 5S RNA (vc5Sa; lower panel). Wt (lanes 1 and 3), *hfq* (lanes 2 and 4) and *rpoS* (lane 5) *V. cholerae* were grown in LB to either log (lanes 1 and 2) or stationary (stat) phase (lanes 3–5). C. Northern blot as in (B), probed with A0943R1 (upper panel) and with vc5Sa (lower panel). RNA was electrophoresed on glyoxal gels. D and E. Northern blots of polyacrylamide gels probed with A10-2, A0943R1 and vc5Sa as above. Wt (lanes 1 and 2) and *hfq* (lanes 3 and 4) *V. cholerae* were grown in LB (lanes 1 and 3) or M63 + maltose and casamino acids (lanes 2 and 4). a, b and c correspond to the long, intermediate and short forms of MicX subsequently determined to be 346, 238 and 188 nt in length.

The sRNA MicX was identified during a bioinformatic search for sRNAs in *V. cholerae* (Livny *et al*., 2005). It was predicted based on the proximity of a putative rho-independent terminator to intergenic sequences conserved between *V. cholerae*, *V. vulnificus* and *V. parahaemolyticus*. The conserved sequence in *V. cholerae* lies immediately downstream of *vca0943* (otherwise known as *malG*, the final gene of a putative operon encoding a maltose transporter), and hence might be expected to be a conserved 3′ UTR (Fig. 1A); however, Northern analysis with a probe complementary to this sequence detected abundant transcripts of < 300 nt, far too short to be the *vca0943* mRNA (Livny *et al*., 2005 and Fig. 1B and D). MicX transcripts have been detected in cells cultured in Luria–Bertani (LB) and in M63 minimal media supplemented with glucose or maltose ± casamino acids, during both log-phase growth and stationary phase (Livny *et al*., 2005; Fig. 1B and D; and data not shown). To date, no conditions have been identified under which wild-type (wt) cells do not produce MicX; however, it appears that MicX abundance may be inversely correlated with growth rate, as transcript levels are slightly reduced when cells are grown in richer media (Fig. 1D). MicX transcripts are dramatically reduced in *V. cholerae* lacking Hfq (Fig. 1B and D), as has been observed for several other regulatory sRNAs (Sledjeski *et al*., 2001; Masse *et al*., 2003; Zhang *et al*., 2003; Opdyke *et al*., 2004). Transcript levels were not altered in *V. cholerae* lacking the alternate sigma factors σ^S^ (Fig. 1B) or σ^E^ (data not shown).

**Fig. 3:**
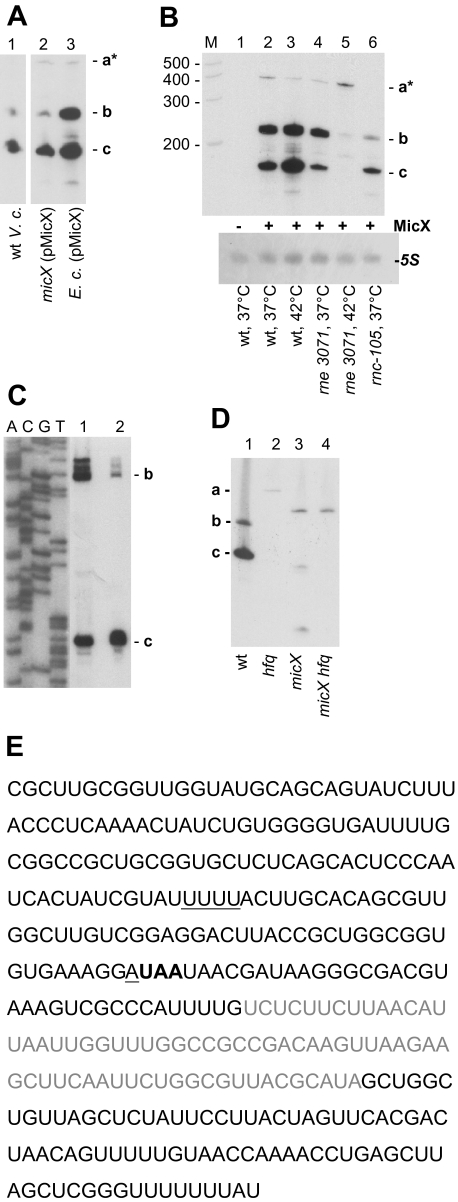
Analysis of MicX endonucleolytic cleavage. Northern blots of polyacrylamide gels probed with A10-2 and vc5Sa (A, B and D) and primer extension analyses (C) were used to monitor MicX processing (a, b and c, as in Fig. 1). A. MicX transcripts in wt *V. cholerae* (lane 1), *V. cholerae micX*_*Δ196−263*_ (pMicX_-2-346_) (lane 2), and *E. coli* DH5α (pMicX_-2-346_) (lane 3). MicX form ‘a*’ is slightly longer than form ‘a’ due to the presence of additional vector-derived sequences. B. *E. coli* strains N3433 (wt, lanes 1–3), N3431 [*rne 3071* (ts); lanes 4 and 5] and AB301-105 (*rnc-105*; lane 6) transformed with pMicX_-2-346_ were cultured with (lanes 2–6) or without (lane 1) arabinose to induce production of MicX. Cells were maintained at 37°C (lanes 1, 2, 4 and 6) or were transferred to the non-permissive temperature of 44°C (lanes 3 and 5). C. Primer extension analyses of RNA from *E. coli* N3433(pMicX_-2-346_) (lane 1) and wt *V. cholerae* (lane 2), run adjacent to a sequencing ladder generated with the same primer. D. Northern blot analysis of MicX RNA in wt (lane 1), *hfq* (lane 2), *micX*_*Δ196−263*_ (lane 3) and *micX*_*Δ196−263*_*hfq* (lane 4) *V. cholerae*. E. Summary of primer extension and sequence analyses of MicX. The full-length MicX sequence is presented. The stop codon of *vca0943* is shown in bold, and cleavage sites (the 5′ ends of MicX forms b and c) are underlined. The region of MicX deleted in *micX*_*Δ196−263*_ is shown in grey.

Multiple forms of MicX were detected on Northern blots: one transcript of approximately the predicted size (c: 189 nt), and two larger, less abundant species (b: 241 nt and a: 346 nt; Fig. 1D). The largest form is often undetectable in wt cells; however, it is the only form ever observed in the *hfq* mutant (e.g. Fig. 1D). As the two larger MicX transcripts were predicted to overlap with *vca0943*, Northern blots were also hybridized with a probe complementary to the final 10 codons of this gene (probe A0943R1; see Fig. 1A). This probe only hybridized to the larger two of the three MicX species, thus providing an approximate position for the 5′ end of the smallest species (also see Fig. 3F). In addition, very faint hybridization to significantly larger RNAs (∼0.9 kb) was detected in some samples; these may correspond to full-length *vca0943* transcripts. The scarcity of these putative mRNAs relative to the abundance of MicX RNA, coupled with the fact that some forms of MicX appear to overlap with *vca0943* mRNA, suggested that MicX might be transcribed from an sRNA-specific promoter, rather than processed from the 3′ end of an mRNA. This hypothesis is consistent with the observation that conditions that alter expression of the operon containing *vca0943* have no detectable effect on the abundance of MicX (data not shown).

**Fig. 2:**
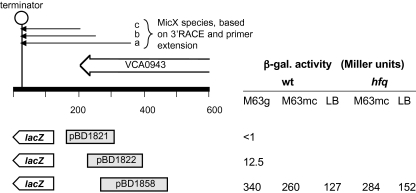
Identification of the MicX promoter. Plasmids pBD1821, pBD1822 and pBD1858 are transcriptional reporter fusions containing DNA fragments (grey boxes) overlapping the 5′ ends of the various forms of MicX (a–c, as in Fig. 1, shown at the top of the figure). Reporter activity was assessed in wt and *hfq V. cholerae* grown to log phase in M63 + glucose (M63 g), M63 + maltose and casamino acids (M63mc), and LB. The numbers shown are an average derived from at least three independent cultures. The 5 and 3′ ends of a, b and c were determined using 3′ RACE and primer extension. *lacZ* transcripts derived from pBD1822 have the same 5′ end as the longest form (a) of MicX.

To confirm the existence of a MicX-specific promoter and to assess whether the three forms of MicX originate from shared or distinct promoters, we generated transcriptional reporter fusions consisting of candidate promoter regions cloned upstream of a promoterless *lacZ* (Fig. 2). High β-galactosidase activity was only generated from a reporter containing sequences extending ∼100 bp upstream of the 5′ end of the largest form of MicX (pBD1858; Fig. 2). Lower activity (> 25× reduced) was generated from a reporter containing sequences upstream of the mid-sized MicX transcript (pBD1822; Fig. 2); however, primer extension analyses revealed that the 5′ end of *lacZ* transcripts derived from pBD1822 was the same as that of the largest form of MicX (data not shown). This reporter contains only 36 nt upstream of the apparent start site; thus, it appears that additional sequences further upstream are required for full MicX promoter activity. No detectable β-galactosidase activity was generated from a reporter fusion containing sequences that spanned the 5′ end of the shortest and predominant form of MicX (pBD1821; Fig. 2). Together, these data suggest that a single promoter drives production of all three forms of MicX, and that the shorter two forms are generated by processing of the longest form. The MicX promoter appears to become less active as nutrient availability is increased (Fig. 2), consistent with the decreased transcript abundance in richer media noted above. No difference in reporter activity was detected between wt and *hfq V. cholerae*, suggesting that decreased MicX abundance in the *hfq* background is not due to altered transcription of MicX.

### Processing of precursor MicX to its predominant forms requires RNase E but not binding to mRNA targets

Previous analyses of sRNA processing and decay have revealed roles for the endoribonucleases RNase E and RNase III (Faubladier *et al*., 1990; Masse *et al*., 2003; Moll *et al*., 2003; Vogel *et al*., 2004; Afonyushkin *et al*., 2005). To assess the contribution of these enzymes to processing of MicX, we expressed a long form of MicX in wt *E. coli* and derivatives lacking these ribonucleases, and then monitored the relative abundance of full-length and truncated MicX. Short transcripts identical in size to endogenous MicX were produced following exogenous expression of MicX in *V. cholerae* and wt *E. coli*, suggesting that similar processing pathways operate in these two organisms and that MicX's mRNA targets (absent in *E. coli*; see below) are not required for sRNA modification (Fig. 3A). Short transcripts were also produced in *E. coli* lacking RNase III (Fig. 3B). However, short transcripts were not detected in a temperature-sensitive RNase E mutant following growth at the non-permissive temperature (Fig. 3B). MicX processing was normal in an isogenic control strain grown at the non-permissive temperature, as well as in the RNase E mutant grown at the permissive temperature. These data strongly suggest that full-length MicX is cleaved by RNase E to generate the short forms of the sRNA. Consistent with this conclusion, primer extension analyses to identify the precise MicX cleavage sites revealed that, like many RNase E recognition sites (McDowall *et al*., 1994), they are AU-rich (Fig. 3C and E). Interestingly, as with the *V. cholerae hfq* mutant, in which only the long form of MicX was detected, *E. coli* lacking RNase E did not accumulate large amounts of the MicX precursor transcript.

Processing of MicX was also monitored in a MicX deletion mutant, which was generated in order to study the biological role of the sRNA. The mutation in this strain removes 68 nt of MicX but does not disrupt the *vca0943* coding sequence or the predicted rho-independent terminator downstream. Functional analyses (described below) indicate that the mutant MicX (MicX_Δ196−263_) no longer regulates target genes, suggesting that domains important for mRNA binding have been removed. However, Northern analyses revealed that the deletion does not prevent MicX processing. Both intermediate and short forms of MicX are generated from the mutant precursor sRNA, although the relative abundance of the three forms differs from that detected in the wt strain (Fig. 3D). These data are consistent with our conclusion that interaction of MicX with its mRNA targets is not a prerequisite for sRNA processing. As with the wt MicX, no processed forms of MicX_Δ196−263_ were observed when *hfq* was also deleted (Fig. 3D).

### Fully processed MicX is more stable than precursor transcripts

**Fig. 4:**
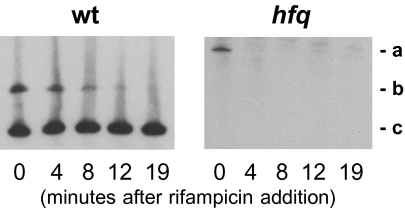
Northern blot analysis of processed and precursor MicX stability. RNA was isolated from early stationary phase (OD_600_ ∼1.1) cultures of wt and *hfq V. cholerae* at varying times following addition of rifampicin (50 μg ml^−1^), and electrophoresed on an acrylamide gel. The blot was hybridized to probe A10-2. The exposure time for the right panel was longer than that for the left panel. Significant overexposure of the left panel (not shown) reveals full-length MicX (form a) in wt cells at t = 0 only. MicX forms a, b and c are as previously described.

The scarcity of full-length MicX, even in the absence of MicX processing, compared with the abundance of the fully processed (189 nt) form, suggested that processed forms of MicX might be significantly more stable. Experiments performed to measure the half-lives of the various MicX species confirmed this supposition. In wt *V. cholerae*, full-length MicX was present only at t = 0, prior to interruption of transcription, and not after 4 or more minutes following addition of rifampicin (data not shown; full-length transcripts were only detected after overexposure of the blot in Fig. 4). A precise half-life cannot be calculated from this result, but it indicates that the half-life of this transcript must be less than 2 min; it is likely that full-length transcripts are rapidly converted into one of the shorter forms in wt cells. In contrast, the intermediate (241 nt) form of MicX is significantly more stable. This transcript was still evident in RNA from wt cells 12 min after addition of a transcription inhibitor, although its abundance was significantly reduced. Furthermore, in the same RNA from wt cells, no notable depletion of fully processed MicX was evident even 19 min following interruption of transcription. Although it is likely that some intermediate MicX was converted to the fully processed form during the course of the experiment – in fact, in some experiments an increase in the abundance of fully processed MicX was observed (data not shown) – and that this might mask some degradation of the fully processed transcript, these data still suggest that fully processed MicX is at least 10× more stable than its precursor transcripts (half-life > 20 min versus half-life < 2 min respectively). It is possible that, as has been observed for other sRNAs (Masse *et al*., 2003), MicX might be less stable when transcription is not inhibited, because its targets could continue to be synthesized; however, there is no reason to suppose that the relative stabilities of the various forms would be altered.

### Identification of MicX mRNA targets

**Fig. 5:**
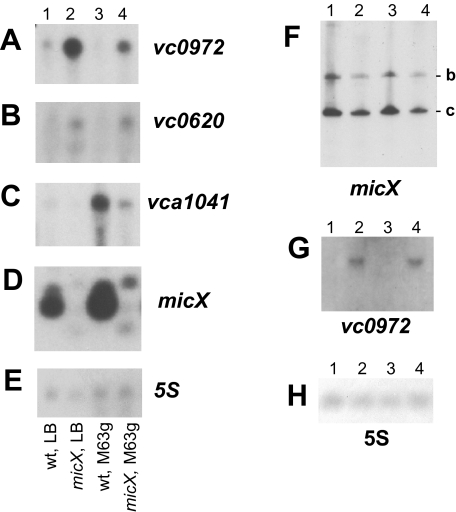
Northern blot analysis of MicX and its potential targets. A–E. RNA from log-phase cultures of wt (lanes 1 and 3) and *micX*_*Δ196−263*_ (lanes 2 and 4) *V. cholerae* grown in LB (lanes 1 and 2) or M63 + glucose (M63g; lanes 3 and 4) was hybridized to probes for the genes indicated for each blot. F–H. RNA from log-phase cultures of wt *V. cholerae* transformed with pAntiMicX_-37-309_ (lanes 1 and 2) or pAntiMicX_103-309_ (lanes 3 and 4) and grown in M63 + maltose and casamino acids, in either the absence (lanes 1 and 3) or presence (lanes 2 and 4) of arabinose, was hybridized to probes for the genes indicated under each blot. RNA was electrophoresed on glyoxal (A–E, G, H) or polyacrylamide (F) gels.

To identify potential mRNA targets of MicX, we compared the transcriptomes of wt and *micX*_*Δ196−263*_ *V. cholerae*, using DNA microarrays. These two strains exhibit comparable growth kinetics both *in vitro* and in the suckling mouse small intestine (data not shown). For the microarray experiments, the strains were grown in M63 media supplemented with glucose and harvested during log-phase growth, a condition in which MicX expression is relatively high. The array analyses revealed that transcript abundance differed consistently (increased or decreased > 1.5×) for only a few genes. Transcripts for two genes were consistently elevated: *vc0972*, which encodes a putative OMP (average increase 2.6×), and *vc0620*, which encodes a putative periplasmic peptide-binding protein for a peptide ABC transporter (average increase 1.7×). Transcripts for only one gene were consistently reduced: *vca1041*, which encodes a putative phosphomannomutase (average decrease 1.8×). Northern analyses confirmed that transcript abundance for all three genes differed in a MicX-dependent fashion, although *vca0141* regulation was not detected if cells were grown in LB instead of M63 (Fig. 5A–E).

To assess the consequences of a more transient MicX deficiency, we also evaluated the effect of antisense MicX on the *V. cholerae* transcriptome. In contrast to experiments with antisense MicA (Udekwu *et al*., 2005), antisense MicX (complementary either to full-length MicX or to its 3′ end) had a relatively minor effect on MicX abundance (Fig. 5F). However, the antisense sRNA still had a significant effect on levels of *vc0972* and *vc0620* transcripts, assessed using Northern blots (Fig. 5G) and/or microarrays (not shown). These data suggest it is possible to inactivate MicX without depleting it. Presumably, binding of MicX to the perfectly complementary antisense RNA occurs more readily than binding to mRNA targets, and hence regulation of mRNAs is abolished. We have not explored why *vca1041* transcript abundance was not altered by the antisense MicX. It is possible that *vca1041* is indirectly, rather than directly, controlled by MicX, and that a longer period of MicX inactivity (i.e. a longer period of antisense MicX induction) is required for this regulation to become manifest.

A bioinformatic approach was also utilized to predict potential MicX targets. Sequences of both full-length MicX and its shortest, most abundant form were used as inputs for the recently described program *TargetRNA* (Tjaden *et al*., 2006; http://snowwhite.wellesley.edu/targetRNA/), which searches for complementarity between sRNAs and sequences surrounding translational start sites. As has been observed for several other sRNAs, *TargetRNA* predicted interactions between MicX and several *V. cholerae* mRNAs whose abundance did not vary (according to microarray analyses) in a MicX-dependent fashion (Table 1); these may be either false positives or true targets whose transcript abundance is not altered by interaction with MicX. However, *TargetRNA* also predicted an interaction between *vc0620* mRNA and both the short and long forms of MicX, suggesting that the MicX-dependent dowregulation of *vc0620* transcripts described above is due to a direct interaction between these RNAs. An interaction between *vc0972* RNA and MicX was not predicted, even when analysis parameters were made less stringent than the default settings.

**Table 1:** MicX mRNA targets predicted by *TargetRNA*.^a^

	Gene product	*P*-value^b^	Microarray transcript ratio (*micX*/wt)^c^
*TargetRNA* prediction: MicX_158-346_			
*vc0253*	IS1004 transposase-related protein	0.0027	n.d.^d^
*vc0870*	IS1004 transposase	0.0027	1.083
*vc0620*	Peptide ABC transporter, periplasmic peptide-binding protein	0.0038	1.69
*vc1769*	DNA methylase HsdM	0.0062	1.052
*vc2259*	Elongation factor Ts	0.0058	1.07
*vc0402*	MSHA biogenesis protein MshL	0.0073	0.904
*vc1848*	Ribosome modulation factor	0.0087	0.954
*TargetRNA* prediction: MicX_1-346_			
*vc0253*	IS1004 transposase-related protein	0.0059	n.d.^d^
*vc0870*	IS1004 transposase	0.0059	1.083
*vc0620*	Peptide ABC transporter, periplasmic peptide-binding protein	0.0080	1.69

a Predictions were generated using default parameters.

b *TargetRNA* output; default threshold is 0.01.

c Average from six experiments.

d Not determined; no gene-specific oligonucleotide is on the array, or may cross-hybridize to *vc0870*.

### Both full-length and processed forms of MicX have regulatory activity

**Fig. 6:**
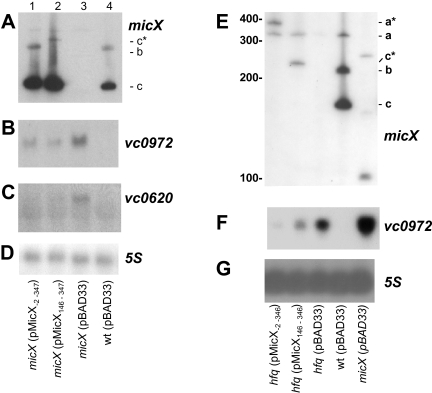
Northern blot analysis of the activity of processed and unprocessed MicX. A–D. RNA from *micX*_*Δ196−263*_*V. cholerae* transformed with pMicX_-2-346_ (lane 1), pMicX_146-346_ (lane 2) or pBAD33 (lane 3), or from wt *V. cholerae* (pBAD33) (lane 4) cultured in M63 + maltose, casamino acids and arabinose was analysed on Northern blots hybridized to probes for the genes noted for each blot. E–G. Northern blots of RNA from *hfq* (pMicX_-2-346_) (lane 1), *hfq* (pMicX_146-346_) (lane 2), *hfq* (pBAD33) (lane 3), wt (pBAD33) (lane 4) and *micX*_*Δ196−263*_ (pBAD33) (lane 5), hybridized to probes for the indicated genes. Strains were grown in LB with arabinose. RNA was electrophoresed on polyacrylamide (A, E) or glyoxal (B–D, F and G) gels. MicX forms a, a*, b and c are as previously described; c* contains 12 nt upstream of the downstream MicX processing site plus additional vector-derived sequences.

As the predominant form of MicX is the fully processed form, we assessed whether this RNA could modulate levels of target mRNAs. Inducible constructs for expression of the short and long forms of MicX were introduced into *micX*_*Δ196−263*_*V. cholerae*, and the abundance of MicX targets was assessed via Northern analysis. Expression of the short and long forms of MicX (Fig. 6A) caused a similar reduction in the levels of *vc0972* and *vc0620* transcripts relative to their abundance in a strain carrying an empty vector (Fig. 6B and C); thus, it is clear that MicX is not inactivated by its RNase E-mediated processing. Given its abundance, it therefore seems likely that processed MicX is responsible for the majority of MicX activity in wt cells. Somewhat surprisingly, target mRNAs were not reduced to the level found in wt *V. cholerae* by either the long or short form of MicX, even though exogenously expressed MicX was more abundant than that found in wt *V. cholerae* (Fig. 6A). It is possible that the presence of mutant (internally deleted) MicX interfered with the activity of the exogenously expressed sRNA.

The previous analyses did not reveal whether the full-length MicX is also active, because the majority of exogenously expressed full-length MicX was processed to shorter forms. To address this question, we assayed mRNA target abundance in *hfq V. cholerae*, in which only unprocessed MicX is detected. Not surprisingly, given the relative scarcity of MicX in the *hfq* background (Fig. 6E, also Figs 1, 3 and 4), *vc0972* mRNA was more abundant in *hfq* than in wt *V. cholerae* (Fig. 6F), but it was not as abundant as in *micX V. cholerae*, suggesting that unprocessed MicX might have regulatory activity. However, because the *hfq* mutation causes a multitude of effects (Ding *et al*., 2004) and, therefore, might influence *vc0972* transcript abundance through MicX-independent processes, we also determined the effect of overexpression of full-length MicX in *hfq V. cholerae*. As with endogenous MicX, no processing of the sRNA was detected in this background, and the abundance of the exogenously expressed transcripts was significantly lower than in wt *V. cholerae* (Fig. 6E); nonetheless, a marked reduction in *vc0972* mRNA was detected in response to full-length MicX expression (Fig. 6F). A reduction in *vc0972* mRNA was also observed when a short form of MicX was expressed in *hfq V. cholerae*. The simplest explanation for these data is that both processed and unprocessed MicX are active and can directly influence the abundance of mRNA targets. These data also suggest that MicX activity is not dependent upon Hfq. It seems likely that Hfq's principal role (with respect to MicX) is to stabilize the sRNA, rather than to mediate its interactions with mRNA targets. Consistent with this idea, we found that endogenous MicX has a half-life of less than 2 min in *hfq V. cholerae* (Fig. 4), even though it does not appear to be depleted via processing to shorter forms. Exogenously expressed short and long forms of MicX accumulated to similar levels (Fig. 6E) and were similarly unstable in this strain background (data not shown); thus, Hfq may bind to and protect multiple forms of the sRNA.

### Mechanism of MicX-dependent mRNA dowregulation

**Fig. 7:**
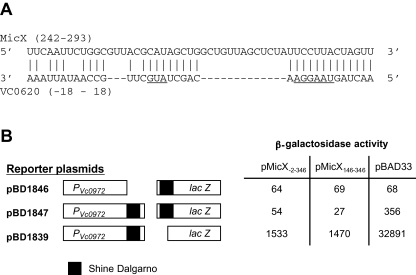
Bioinformatic and functional characterization of mRNA sequences likely to interact with MicX A. Pairing between MicX and *vc0620* mRNA according to *TargetRNA.* MicX is numbered relative to the start of transcription; *vc0620* RNA is numbered relative to the start of translation. Vc0620s start codon and probable ribosome binding site are underlined. B. Schematic representation of *vc0972::lacZ* reporter fusions and their activity (in Miller units) in the presence or absence of MicX. Numbered relative to the start of translation, pBD1846 contains nt −402 to −26 of *vc0972*, and pBD1847 and pBD1839 contain nt −402 to 55. Primer extension analyses (not shown) suggest that transcription initiates at nt −137. Translation from pBD1839 transcripts utilizes the *vc0972* ribosome binding site. Translation from pBD1847 utilizes only the *lacZ* ribosome binding site, as there are two stop codons downstream of the *vc0972* ribosome binding site. Cells were cultured in LB + arabinose. The numbers shown are an average obtained from at least three independent cultures.

*TargetRNA* analyses suggest that MicX pairs with the start codon and Shine Dalgarno sequence of *vc0620* mRNA (Fig. 7A), presumably thereby inhibiting translation and promoting mRNA instability, as has been observed for several other sRNA/mRNA pairs. To assess whether MicX might interact in a similar fashion with *vc0972* mRNA, we generated a series of *vc0972::lacZ* reporter fusions and assessed their activity in the presence and absence of MicX. We found that a transcriptional fusion containing nt −402 to −26 of *vc0972* (relative to the translational start site) was not affected by exogenous expression of the short or long form of MicX in *E. coli* (pBD1846; Fig. 7B). In contrast, transcriptional and translational fusions containing nt −402 to 55 (relative to the translational start site) were 7- to 20-fold less active in the presence of MicX than in its absence (pBD1847 and pBD1839; Fig. 7B). These data confirm our finding (Fig. 6A and C) that the short form of MicX has regulatory activity, and suggest: (i) that transcription of *vc0972* is not altered by the presence of MicX; (ii) that regulation of *vc0972* by MicX is mediated by a region near or overlapping with the translational start site; and (iii) that regulation of *vc0972* by MicX is not dependent on additional *V. cholerae*-specific factors and, by analogy to other sRNA/mRNA pairs, is likely to be due to a direct interaction between the two RNAs. In addition, the observation that a transcriptional fusion that contains nt −402 to 55 of *vc0972* (i.e. a fusion that contains the *vc0972* RBS but uses a downstream RBS for β-galactosidase translation) is regulated by MicX suggests that MicX binding destabilizes the target mRNA, rather than simply interfering with its translation. As the first event in mRNA degradation is often rate-limiting (Kushner, 2002), cleavage of *vc0972* sequences in the reporter transcript probably promotes degradation of the remaining transcript and thereby reduces reporter activity. A reduction in *vc0972::lacZ* transcripts in response to MicX has been confirmed for pBD1839 (data not shown). Similar mRNA destabilization presumably accounts for the MicX-dependent dowregulation of *vc0972* transcript abundance observed in *V. cholerae*.

## Discussion

We have characterized MicX, a *V. cholerae* sRNA initially identified through bioinformatic analyses and formerly known as A10. The DNA encoding primary transcripts of MicX overlaps with the *vca0943* (*malG*) open reading frame (ORF), but transcripts for these two genes originate at distinct promoters. The vast majority of MicX transcripts are processed, in an RNase E-dependent fashion, to a shorter form that contains only a few nucleotides of the *malG* coding sequence. Processed MicX retains biological activity; bioinformatic and functional analyses indicate that it dowregulates the transcript abundance of *vc0972* (which encodes a putative OMP) and *vc0620* (which encodes a putative periplasmic component of an ABC transporter). Full-length MicX also appears to have biological activity; however, because unprocessed transcripts are unstable and relatively rare, they probably contribute minimally to MicX-mediated regulation, at least in wt cells.

Processing of an sRNA precursor to an abundant and active form is uncommon among sRNAs characterized to date, but not entirely unprecedented. For example, DsrA and GadY are each expressed from a single promoter, yet multiple active sRNA species can be detected for both (Majdalani *et al*., 1998; Repoila and Gottesman, 2001; Opdyke *et al*., 2004). Like processing of MicX, processing of DsrA is altered in a strain lacking RNase E (Moll *et al*., 2003). However, unlike the case with DsrA (and with other RNAs that are substrates for this nuclease, such as RyhB and SsrA), MicX is less abundant in a strain lacking RNase E than in a wt strain. Full-length MicX is more prevalent in the *rne* background than in the wt background, but these transcripts are scarce in comparison with the level of processed transcripts seen in wt cells. It is clear that full-length MicX is relatively unstable; it seems likely that in the absence of RNase E-mediated processing, it is degraded by other nucleases.

Our data suggest that both full-length and processed MicX have biological activity; consequently, it is not clear why processing occurs. It does not appear to be dependent upon binding to mRNA targets, as it is detectable when MicX is expressed in *E. coli*, as well as when regions necessary for its activity (and presumably its pairing with targets) are deleted. The low accumulation of full-length MicX, even in the absence of processing and targets, as well as the notably longer half-life of the processed species, both indicate that MicX is more stable following processing. Thus, one potential explanation for processing is to make it more likely that degradation of MicX is coupled to that of a target mRNA, rather than being a result of non-specific nuclease cleavage.

The role of Hfq with respect to MicX remains somewhat ambiguous. Exogeously expressed short and long forms of the sRNA have regulatory activity in cells lacking Hfq, suggesting that binding of the sRNAs to mRNA targets can occur in its absence. However, in the absence of Hfq, neither endogenous nor exogenously expressed MicX appear to be processed, suggesting that processing of MicX may require Hfq as well as RNase E. In support of this possibility, we have observed that full-length MicX transcripts are typically more abundant in *hfq V. cholerae* than in wt cells. As in wt cells, they are quite unstable; however, their increased accumulation may reflect the elimination of processing as a means of transcript depletion. Given the role proposed above for RNase E in processing of MicX, coupled with the absence of reports of Hfq-mediated RNA cleavage, it seems unlikely that Hfq is directly responsible for cleavage of this RNA. Instead, we hypothesize that Hfq, which has been shown to alter the structure of other RNA species, may promote folding of MicX into a structure susceptible to RNase E cleavage.

An alternative explanation for the absence of processed MicX in *hfq V. cholerae* is that processed transcripts may be extremely unstable in this genetic background. Analyses of other sRNAs have revealed that Hfq can play a dramatic role in transcript stabilization (Zhang *et al*., 2003), and it seems likely that Hfq stabilizes MicX as well. However, as we have never observed any processed MicX in the absence of Hfq, even after extended exposure of Northern blots, there is currently no evidence to suggest that it is formed. Furthermore, exogenously expressed short and long forms of MicX accumulate to comparable (albeit low) levels in Hfq-deficient cells (Fig. 6E). Although this is consistent with Hfq being needed to stabilize both forms of MicX, it also suggests that preferential destruction of processed MicX does not occur in these cells. Consequently, if Hfq is not required for RNase E-mediated cleavage of MicX, as proposed above, then it is not clear why some low levels of processed transcripts are not detected.

It remains to be seen whether transcripts for *vca0943*, which should contain all of the sequences that govern MicX cleavage, are also processed by RNase E, and if so, what the biological consequences of such cleavage are. From a functional standpoint, such processing appears unlikely, as it would remove the stop codon and (depending upon the cleavage site) an additional 50 nucleotides of coding sequence. Also, preliminary studies did not reveal unusually long transcripts for *vca0943* in *hfq V. cholerae*, where processing presumably would not occur (data not shown). It is possible that the additional sequences in *vca0943* transcripts preclude formation of secondary structures needed for RNase E-mediated cleavage, or that translation impedes access by nucleases. However, it is also possible that processed transcripts are formed but are degraded (e.g. due to unsuitability for translation), and hence have not been detected.

Microarray analyses enabled identification of two genes – *vc0972* and *vc0620* – whose transcript abundance is consistently modulated by MicX. Bioinformatic and experimental approaches suggest that regulation of both is a consequence of direct interactions between MicX and sequences near the translational start sites of the mRNAs, as has also been reported in analyses of several *E. coli* sRNAs. Such sRNA:mRNA pairing can inhibit ribosome binding and hence translation (Moller *et al*., 2002b; Udekwu *et al*., 2005), and consequently is expected to render target transcripts more susceptible to nuclease digestion. Analyses of *vc0972::lacZ* reporter fusions suggest that a comparable paradigm applies to MicX-dependent regulation. Reporter transcripts that contained *vc0972*'s apparent RBS yielded reduced β-galactosidase activity following expression of MicX, even if the RBS was not needed for synthesis of β-galactosidase (e.g. from pBD1847). These results are consistent with MicX-dependent mRNA destabilization. It remains to be seen if pairing of MicX to its targets also results in degradation of the sRNA, as has been proposed for other sRNAs (Masse *et al*., 2003; Vogel *et al*., 2004). If such degradation occurs, one might expect elevated MicX levels in a strain lacking the target. We did not observe increased MicX following deletion of *vc0972* (including the 5′ UTR) (data not shown); however, it is possible that the abundance of MicX relative to *vc0972* transcripts results in degradation of only a small and consequently undetected fraction of MicX.

Although MicX clearly functions as a regulator of *vc0972* and *vc0620*, its overall contribution to *V. cholerae* physiology remains to be determined. Neither of the two targets has been well characterized; hence the consequences of their deregulated expression are unknown. Furthermore, because MicX is expressed under all conditions tested to date and does not appear to be dependent upon alternate sigma factors (unlike many other sRNAs, which have been found to be induced by particular stimuli, generally stresses), conditions that favour or necessitate expression of the target genes have not been identified. Deletion of MicX and the ensuing overexpression of its targets does not appear to be detrimental to *V. cholerae*, as no notable difference in growth between wt and *micX* strains has been observed either *in vitro* or in the suckling mouse intestine. Thus, MicX insufficiency does not appear to account for or contribute to the severe colonization defect observed for a *V. cholerae hfq* mutant (Ding *et al*., 2004).

It is noteworthy that MicX is a regulator of OMP production, as to date OMP-encoding genes appear to be among the most frequent targets of sRNAs in *E. coli* (reviewed in Guillier *et al*., 2006). Our analysis of the transcriptome of *hfq V. cholerae* (Ding *et al*., 2004) suggests that several additional OMPs in *V. cholerae* may also be regulated by sRNAs [e.g. *vc1854* (*ompT*), *vc2213* (*ompA*) and *vca1028* (*ompS*)]. However, despite the thematic similarity between *V. cholerae* and *E. coli*, there is no detectable homology between MicX or its targets and *E. coli* sequences. In particular, blastn analyses did not identify MicX homologues (*E* < 2 × 10^−8^) in species other than *V. parahaemolyticus* and *V. vulnificus*. Furthermore, the position of *micX* in the *V. cholerae* genome (overlapping with an upstream ORF) differs from those of *E. coli*'s sRNA OMP regulators, which in several instances are upstream of, and divergently transcribed from, a non-target OMP-encoding gene (e.g. adjacent gene pairs *micF/ompC* and *micC/ompN*). blastn analyses of MicX's targets (estimated as −100 to +100 of each gene, relative to their translational start sites) similarly detected related sequences (E < 10^−1^) only in the *Vibrionaceae* and in *Photobacterium profundum*, until recently a member of the *Vibrionaceae.* It will be interesting to explore whether subtle similarities remain to be detected between the sRNA regulators of *V. cholerae* and *E. coli* OMPs, and why OMPs are apparently overrepresented among sRNA targets.

## Experimental procedures

### Bacterial strains and culture conditions

All *V. cholerae* strains used in this study are derivatives of the sequenced clinical isolate, N16961 (Heidelberg *et al*., 2000). However, in sequencing plasmids and strains derived from N16961, we noted that 1 nt within the 3′ UTR of *vca0943* (and within MicX) is consistently absent from our clones; the MicX sequence presented in Fig. 3 is that from our strains. The *E. coli* strains DH5α, N3433 (Goldblum and Apirion, 1981), N3431 [*rne 3071* (ts); Miczak and Apirion, 1993] and AB30-105 (*rnc-105*; Apirion and Watson, 1975) were used for analyses of MicX processing. Assays of β-galactosidase activity in *E. coli* were performed in derivatives of strain BW27784 (Khlebnikov *et al*., 2001). Bacteria were cultured either in LB or in M63 supplemented with glucose (0.2%) or maltose (0.2%) ± casamino acids (0.1%) as indicated; they were incubated at 37°C unless otherwise noted. Antibiotics were used at the following concentrations: streptomycin, 200 μg ml^−1^; ampicillin, 100 μg ml^−1^; and chloramphenicol, 20 μg ml^−1^ (*E. coli*), 5 μg ml^−1^ (*V. cholerae*). Arabinose (0.02%) was added to induce the promoter P_BAD_.

### Strain and plasmid construction

*lacZ* was deleted from all *V. cholerae* strains using the allele exchange vector pJL1 (D.T. Beattie, unpublished), a derivative of p6891MCS (Butterton *et al*., 1995). N16961 *hfq* was constructed as described (Ding *et al*., 2004). N16961 *micX*_*Δ196−263*_ (a.k.a. BD1820, containing a deletion of nt 196–263 of *micX*, relative to the start of transcription) and M16961 *hfq micX*_*Δ196−263*_ (a.k.a. BD1855) were generated using pBD1816 and standard allele exchange procedures (Donnenberg and Kaper, 1991). Briefly, pBD1816 was transferred by conjugation from the donor strain SM10λpir to a *V. cholerae* recipient, where it integrated. Exconjugants were then subject to counterselection on sucrose plates to identify strains that no longer contained the plasmid backbone, and were further screened to identify colonies retaining mutant *micX*. The insert for pBD1816 was generated by overlap extension polymerase chain reaction (PCR), using the primers A10-4: GCTCTAGAGGATTTCACGCTTCTGTTATGG, A10-5: GCCCATTTTGGCTGGCTGTTAGCTCTATTCC, A10-6: AACAGCCAGCCAAAATGGGCGACTTTACG, and A10-7: GCTCTAGAGGGGTACTTTGAAACCATCG; it was digested with XbaI and ligated into the counterselectable (*sacB*) allele exchange vector pCVD442 (Donnenberg and Kaper, 1991).

The reporter fusions for assaying MicX promoter activity (pBD1858, pBD1822 and pBD1821) were made by ligating PCR-amplified *V. cholerae* sequences into the reporter vector pCB182 (Schneider and Beck, 1986). pBD1858 contains *V. cholerae* sequences amplified with A10-11: GCTCTAGAATAGTGATTGGGAGTGCTGAG and A10-12: CGGGATCCTGCTACTTCCATTGTCTGTGC, digested with XbaI and BamHI, and ligated to pCB182. The reporter fusion pBD1822 was generated similarly, using primers A943F1: CGGGATCCTGGCGTCACTGCTGCTTTC and A10-9: GCTCTAGAAGTCCTCCGACAAGCCAACG. Likewise, pBD1821 was generated using primers A10-8: CGGGATCCTTTTACTTGCACAGCGTTGG and A10-3: GCTCTAGAAAGAAGAGACAAAATGGGCG.

A long form of MicX was exogenously expressed using pMicX_-2-346_ (a.k.a. pBD1876), which consists of *V. cholerae* DNA amplified with A10-13: CCCAAGCTTAACCTGTTTTGGCGGAGACG and A10-14: GCTCTAGACACGCTTGCGGTTGGTATGC, cloned into the TA cloning vector pCRII (Stratagene), released with XbaI, and ligated to pBAD33 (Guzman *et al*., 1995). A short form of MicX was exogenously expressed using pMicX_146-346_ (a.k.a. pD1876), which was constructed similarly, using the primers A10-15: GCTCTAGACGGTGTGAAAGGATAATAACG and A10-13. Antisense MicX was produced using pAntiMicX_-37-309_ (a.k.a. pBD1841), which consists of *V. cholerae* DNA amplified with A10-2: AAAAACTGTTAGTCGTGAAACTAGTAAGGA and A943F1, cloned into the TA cloning vector pCRII, released with KpnI and PstI, and cloned into pBAD33. Similarly, antisense transcripts complementary to the two shorter forms of MicX were generated with pAntiMycX_103-309_ (a.k.a. pBD1842), using the primers A10-2 and A10-8.

The reporter fusions for assaying *vc0972* transcription and translation (pBD1846, pBD1839 and pBD1847) were made by ligating PCR-amplified *V. cholerae* sequences into pCB182. pBD1846 contains sequences amplified with vc0972F3: CGGGATCCTGCTCAGAAACAACTCGCC and vc0972R3: GCTCTAGAGCTGTTTTTTGAGTTTCTCCC. The reporter fusion pBD1839 contains sequences amplified with vc0972F3 and vc0972R4: GGAATTCGTTGACGCCATCGCTACTGC. Finally, pBD1847 contains sequences amplified with vc0972F3 and vc0972R4.

### RNA analyses

RNA was harvested from log-phase cultures unless otherwise noted. For analyses using *E. coli* RNase mutants, heat shock (44°C) was performed for 20 min, and cell cultures were frozen in dry ice/EtOH prior to centrifugation. RNA was extracted from cell pellets using Trizol (Invitrogen), then treated with DNase I (Qiagen). For Northern blots, RNA was electrophoresed on glyoxal (Ambion) or 6% polyacrylamide gels, then transferred to Bright Star Plus nylon membranes (Ambion). RNA integrity was confirmed via assessment of EtBr-stained rRNA bands on agarose gels. Blots were hybridized to ^32^P-labelled oligonucleotide probes in ULTRAhyb-Oligo (Ambion) at 40°C and washed according to the manufacturer's instructions. Probes for MicX were A10-2 and A0943R1: TTATTCCTTTCACACCGCCAGCGGTAAGTCC. MicX target probes were VC972R: ACCGTATTCCAGACCTACACGTACGAAACC, VC0620R1: GTTGTAGCCGCTGTTCCAGTAAGTGAATGG, and A1041R1: TAGGTTTTGATGCCGAGTGAAGTCAGCACG.

For primer extension analyses, RNA was reverse transcribed with MonsterScript (Epicentre Biotechnologies) at 60°C according to the manufacturer's instructions. Primers were A10-9 (for 5′ end mapping) and A10-16: AGCTAACAGCCAGCTATGC (for cleavage site mapping). For 3′ end mapping of MicX, RNA was polyadenylated using *E. coli* Poly(A) polymerase (Ambion), reverse transcribed using the primer 3′RACE1: TCACGACTCACTATAGGATCCTTTTTTTTTTTTN, amplified with the primers A10-5 and 3′RACE2: TCACGACTCACTATAGGATCC, and cloned into the TA cloning vector pCRII for sequencing.

### β-Galactosidase assays

Assays were performed basically as described (Miller, 1992).

### Microarray analyses

For comparison of the N16961 and N16961 *micX*_*Δ196−263*_ transcriptomes, cells were grown in M63 + glucose to OD_600_∼0.3; three independent pairs of RNAs were generated. For comparison of the N16961(pBAD33) and N16961(pAntiMycX_103-309_) transcriptomes, cells were grown in M63 + maltose and casamino acids, and arabinose was added for the final 30 min of growth; two independent pairs of RNAs were generated. Duplicate experiments were performed with each pair of RNAs. Microarray hybridization and analysis was performed as described (Ding *et al*., 2004), except that hybridization was extended to ∼40 h. Genes were counted as differentially regulated if the ratio of transcript abundance was either > 1.5 or < 0.66 for five of six, or four of four experiments.

#### 

##### Bioinformatic analyses

Potential MicX targets were identified using *TargetRNA* (http://snowwhite.wellesley.edu/targetRNA/). Results presented were generated using the program's default parameters. blast targets were identified only among bacterial species. blast analysis of MicX was performed using the fully processed form of MicX, as the unprocessed sRNA has widespread homology among bacteria, due to its overlap with *malG*.
